# A Guinea Pig Model of Pediatric Metabolic Dysfunction-Associated Steatohepatitis: Poor Vitamin C Status May Advance Disease

**DOI:** 10.3390/nu17020291

**Published:** 2025-01-15

**Authors:** Kamilla Pedersen, Ankita Poojari, Simone Frederikke Colberg, Stine Marguerite Mechernsee, Jo Frøkjær Iversen, Romain Barrès, Jens Lykkesfeldt, Pernille Tveden-Nyborg

**Affiliations:** 1 Section of Preclinical Disease Biology, Department of Veterinary and Animal Sciences, Faculty of Health and Medical Sciences, University of Copenhagen, 1870 Frederiksberg, Denmark; kamilla.pedersen@sund.ku.dk (K.P.); simonecolberg@yahoo.dk (S.F.C.); stinemechernsee@outlook.com (S.M.M.); jopl@sund.ku.dk (J.L.); 2 Thomas J. Long School of Pharmacy, University of the Pacific, Stockton, CA 95211, USA; apoojari@pacific.edu; 3 Novo Nordisk Foundation Center for Basic Metabolic Research, University of Copenhagen, 2200 Copenhagen, Denmark; jo.iversen@sund.ku.dk (J.F.I.); barres@sund.ku.dk (R.B.); 4 Institut de Pharmacologie Moléculaire et Cellulaire, CNRS and Université de Nice Côte d’Azur, 06560 Valbonne, France

**Keywords:** steatohepatitis, guinea pig MASH, animal models, children, juvenile, portal inflammation, fibrosis, vitamin C, DNA hydroxymethylation

## Abstract

**Background/Objectives:** Children and teenagers display a distinct metabolic dysfunction-associated steatohepatitis (MASH) phenotype, yet studies of childhood MASH are scarce and validated animal models lacking, limiting the development of treatments. Poor vitamin C (VitC) status may affect MASH progression and often co-occurs with high-fat diets and related metabolic imbalances. As a regulator of DNA methylation, poor VitC status may further contribute to MASH by regulating gene expression This study investigated guinea pigs—a species that, like humans, depends on vitC in the diet—as a model of pediatric MASH, examining the effects of poor VitC status on MASH hallmarks and global DNA methylation levels. **Methods:** Sixty-two juvenile guinea pigs were exposed to a high-fat diet for 16 weeks. **Results:** Juvenile guinea pigs exhibited hepatic histopathology representative of pediatric MASH, confirmed by portal inflammation and fibrosis. Consistent with pediatric MASH, juvenile guinea pigs displayed increased lobular and portal inflammation (*p* < 0.05 and *p* < 0.0001, respectively) but less steatosis (*p* < 0.001) compared to adults. Compared to the controls, the guinea pigs deprived in VitC showed lower body weight (*p* < 0.01), higher expression of hepatic inflammatory genes (*p* < 0.05), and a lower global hydroxymethylcytosine to methylcytosine ratio in the high-fat groups (*p* < 0.05). **Conclusions:** Our study validates guinea pigs as a model of pediatric MASH and suggests that VitC contributes to an altered gene expression signature through the regulation of DNA hydroxymethylation. We postulate that nutritional co-deficiencies in MASH, such as low VitC, may accelerate disease progression and deserve further attention.

## 1. Introduction

Metabolic dysfunction-associated steatotic liver disease (MASLD)—previously termed non-alcoholic fatty liver disease (NAFLD)—affects 5–10% of all children and adolescents, leading to compromised liver function, serious co-morbidities such as cardiovascular disease, and increased mortality in adulthood [1,2,3,4,5]. Importantly, the pediatric form of metabolic dysfunction-associated steatohepatitis (MASH) seen in children and adolescents displays a more aggressive histopathology and concourse than what is reported for adults [5,6,7]. For example, though they had a comparable BMI and metabolic profile, adolescent MASH patients displayed increased liver damage and hepatic fibrosis as well as increased plasma levels of C-reactive protein compared to their adult counterparts, indicating this as a more severe disease phenotype with increased negative systemic effects [6].

The MASH histopathology seen in children and adolescents can be divided into two subtypes (Type I and II). Both exhibit the histopathological features of MASH: the presence of hepatocellular steatosis (lipid vesicles in >5% of hepatocytes) and inflammation, progressing to include hepatic fibrosis. Type I is characterized by inflammatory foci distributed throughout the hepatic lobule, with fibrosis originating around the central vein and zone 3 sinusoids, and the presence of ballooning hepatocytes and Mallory–Denck bodies, corresponding to the MASH histopathology seen in adults [8]. Type II, on the contrary, displays a characteristic pattern of portal and periportal pathology with inflammation predominantly located around the portal areas (zone 1), which is also where the developing fibrosis originates, and there are very few ballooning hepatocytes [7,8]. In more than two-thirds of pediatric MASH cases, Type II or an overlap between Type I and II are reported, supporting that pediatric MASH may be regarded as a specific disease entity or perhaps an immature state of the disease that may progress to Type I in adulthood [4,7,8,9,10,11,12,13]. As portal pathology is associated with poorer metabolic outcomes and advanced hepatic fibrosis, this could partially explain the increased disease severity in children and adolescents [12,14].

Due to practical considerations and ethical limitations, clinical data and research efforts in children affected by MASH are scarce [2]. This leaves a significant gap in our current understanding of disease mechanisms in this population. In children, even more than in adults, surrogate diagnostic criteria (e.g., alanine aminotransferase (ALT) and ultrasound) are preferred over invasive liver explorations, so a critical mass of histology has not been reached for disease characterization, and consequently, the identification of the mechanisms at play [2,15,16,17]. Classical experimental animal models suffer from a lack of translational validity, impairing progress in the field. Murine, porcine, and rabbit models have been explored [18,19,20], and while MASH can be established in young animals of these species, at best, they only display selected aspects of pediatric MASH and fall short of demonstrating distinct differences from their adult counterparts. Uniquely, guinea pigs closely mimic human MASH histopathology—including progressive hepatic fibrosis—and the associated liver transcriptome when exposed to a high-fat diet [21,22]. In addition, guinea pigs are a precocial species and feed independently shortly after birth, enabling exposure to a high-fat diet at an early age, opposed to the immature pups from, e.g., mice and rats (altrecian species) that require substantial postnatal development before weaning. Consequently, the guinea pig MASH model holds promise as a model for studying pediatric MASH with a superior translational potential.

Vitamin C (VitC) deficiency is linked to several metabolic lifestyle-associated diseases including obesity, cardiovascular disease, and MASLD/MASH in humans, and VitC status may additionally be directly affected in disease [23,24,25,26,27]. Though scurvy (a potentially lethal state of severe and long-term VitC depletion) is rarely encountered today, hypovitaminosis C (plasma levels < 28–23 µM) and VitC deficiency (<11 µM in plasma) [28,29] are not uncommon. Some studies report VitC deficiency in more than 20% of children, though these are subjected to vast variations including geographical and socio-economic factors [23]. Given their rapid development and growth, children may be more susceptible to deficits in micronutrients such as VitC [30,31]. In addition, the intake of an adverse high-fat diet and subsequent obesity may reduce VitC status [24,26,32]. Low levels of VitC have been linked to MASLD, with plasma VitC shown to be inversely associated with MASLD severity in adults [25,33]. In children, reduced VitC intake is linked to an increased number of ballooning hepatocytes [34]. Along with its actions as antioxidant, VitC is a co-factor in various enzymatic reactions and is linked to carnitine synthesis and epigenetic regulation of gene expression [35,36]. VitC is a co-factor for ten–eleven translocation methyl cytosine dioxygenases (TET enzymes), which facilitate the demethylation of cytosines within DNA and are thought to contribute to the epigenetic regulation of gene expression [36]. In addition, VitC is an essential cofactor in collagen maturation and contributes to the formation of the fibrogenic extracellular matrix and possibly also hepatic fibrosis [37,38]. As guinea pigs, like humans, lack endogenous VitC synthesis and depend on dietary intake [39], they represent an appropriate model for investigating the impact of low VitC intake on human health and disease, including pediatric MASH.

This study establishes a guinea pig model of pediatric MASH and explores the potential exacerbating effects of a chronic state of non-scorbutic VitC deficiency on MASH development in juvenile guinea pigs.

## 2. Materials and Methods

### 2.1. Animal Experiments

The animal experiments adhered to the European legislation on animal experimentation (Directive 2010/63/EU), gained approval from the Danish Animal Experiments Inspectorate under license number 2018-15-0201-01591, gained approval from the Institutional Ethics Committee of University of Copenhagen, Faculty of Health and Medical Sciences (protocol code P20-230 08.12.2020), and are reported in alignment with the ARRIVE guidelines [40].

**Figure 1:**
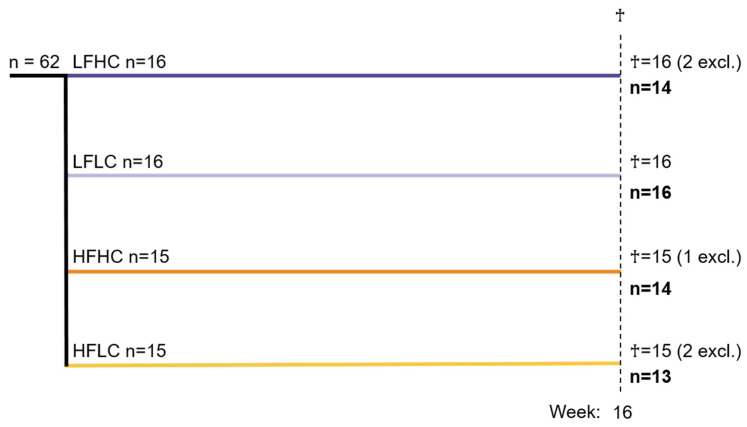
Study design. Male guinea pigs (n = 62) aged 1–2 weeks were block-randomized based on weight into four different diet groups: low-fat high-VitC (LFHC), low-fat low-VitC (LFLC), high-fat high-VitC (HFHC), high-fat low-VitC (HFLC). They continued their study diet for 16 weeks before being euthanized (†). Five guinea pigs (two from LFHC, one from HFHC and two from HFLC) were excluded from the study, as described in the section “Excluded animals”.

Upon arrival, 62 male guinea pigs (Charles River Laboratories, Lyon, France) weighing 150–200 g, corresponding to 1–2 weeks of age, had subcutaneous microchips implanted (E-vet, Haderslev, Denmark). The guinea pigs were randomized into four weight-stratified groups that initially received a low-fat control diet high in VitC (LFHC). Following one week of acclimatization, experimental diets were gradually introduced over a period of five days, establishing the following groups: low-fat high-VitC (LFHC; n = 16; 3.8% fat, 0% sucrose, 0% cholesterol, 1500 mg VitC/kg feed); low-fat low-VitC (LFLC; n = 16; 3.8% fat, 0% sucrose, 0% cholesterol, 50 mg VitC/kg feed); high-fat high-VitC (HFHC; n = 15; 20% fat, 15% sucrose, 0.35% cholesterol, 1500 mg VitC/kg feed); high-fat low-VitC (HFLC; n = 15; 20% fat, 15% sucrose, 0.35% cholesterol, 50 mg VitC/kg feed) (Figure 1) (details of feed composition can be found in Supplementary Table S1). All groups were fed ad libitum. To prevent auto-oxidation and preserve palatability, the feed was stored at −18 °C and thawed daily. The diets were chow-based (Ssniff Spezialdiäten GmbH, Soest, Germany), with VitC added in the form of phosphorylated ascorbate (Stay-C) and confirmed via postproduction analyses (Ssniff Spezialdiäten GmbH, Soest, Germany). The high VitC dose of 1500 mg/kg feed ensured VitC saturation [41]. The VitC concentration in HFHC was obtained via titration of feed containing 1830 mg VitC/kg with feed containing 143 mg VitC/kg, while the VitC concentration in HFLC was obtained by mixing feed containing 143 mg VitC/kg feed with feed containing no VitC. The VitC concentrations in the LF diets were matched to the feed intake in the high-fat groups throughout the study, ensuring that LFHC and LFLC consumed the same amount of VitC as HFHC and HFLC, respectively. Group size calculations were based on histopathological variances from previous studies in adult guinea pigs, a power of 0.80, a significance level < 0.05, and an effect size of 30% was considered biologically relevant. As there are no previous studies of histopathological MASH endpoints in juvenile guinea pigs, the group sizes were increased to ensure sufficient data.

The guinea pigs were group-housed in floor pens with wood shavings, shelters, environmental enrichment, and ad libitum access to hay and water. Feed intake was measured bi-weekly by subtracting the amount of feed remaining in the feeding bins from the amount served the previous day. The housing temperatures ranged from 20–24 °C, with a 12 h light–dark cycle. All handling and management of the animals was performed by experienced animal caretakers and veterinarians qualified to work with experimental animals and specifically experienced with guinea pigs. Animal welfare was monitored daily, while body weights were recorded twice a week for the first eight weeks and once a week for the next eight weeks. Humane endpoints included body weight loss of 20%. This did not occur. However, as weight stagnation is an early sign of severe VitC deficiency (proceeding scurvy), this was also included as a specific point of awareness. In case of weight stagnation in two consecutive weighings, VitC supplementation was initiated, and the animals were monitored closely to ensure weight gain. For two weeks (experimental weeks 12–14), the HFLC and LFLC groups had their dietary VitC concentration increased to 100 mg VitC/kg feed due to weight stagnation in the HFLC group.

Following 16 weeks on the experimental diets, all of the guinea pigs were euthanized over the course of five days in a randomized and blinded manner as previously described [18]. In short, the animals were semi-fasted overnight (access to hay and water) before being pre-anesthetized with 1.25 mL/kg body weight of 10-fold diluted Zoletil mixture (125 mg tiletamin and 125 mg zolazapam (Zoletil 50 vet; Virbac, Nice, France) with 200 mg xylazine (Narcoxyl vet 20 mg/mL; Intervet International, Boxmeer, The Netherlands) and 7.5 mg butorphanol (Torbugesic vet 10 mg/mL; Scanvet, Fredensborg, Denmark)). After 15–20 min, the animals were placed on inhalation anesthesia with 3–5% isoflurane (Isoba vet 100%, Intervet International, København, Denmark). When inter-digital reflexes had disappeared, intracardial blood was collected, followed by euthanization by decapitation.

### 2.2. Excluded Animals

Towards the end of the study, some guinea pigs began to show aggressive behavior, resulting in a total of five guinea pigs that had to be isolated from their group (two from LFHC, one from HFHC, and two from HFLC) for the last 1–2 weeks before euthanasia. According to Danish legislation, guinea pigs can only be single housed for shorter periods of time due to risk of isolation stress. This was confirmed in the present study by a reduced feed intake during single housing. As this was reflected in plasma and liver VitC levels, these animals were excluded from the study.

### 2.3. Plasma Samples

Intracardial blood was collected in 10 mL syringes flushed with K_3_-EDTA and distributed into two K_3_-EDTA-coated vacutainers. The samples were centrifuged at 2000× *g* for 4 min at 4 °C, after which the plasma was collected. For analysis of ALT, aminotransferase (AST), gamma-glutamyl transferase (GGT), triglycerides (TG), and total cholesterol (TC), the plasma was stored at −80 °C until analysis was performed according to the manufacturer’s instructions using an Atellica^®^ CH 930 Analyzer (Siemens, Erlangen, Germany). For analysis of VitC, the plasma was stabilized in 10% metaphosphoric acid (MPA) and stored at −80 °C until analysis via electrochemical detection using high-performance liquid chromatography (HPLC) as previously described [42]. One animal from LFHC had to be excluded from the plasma VitC analysis due to technical issues.

### 2.4. Liver Samples

The entire liver was removed immediately after euthanasia, rinsed in cold phosphate-buffered saline (PBS) (140 mM NaCl, 10 mM phosphate, 3 mM KCl, pH 7.4, Millipore, Billerica, MA, USA), weighed, and photographed. From the left lateral liver lobe, samples for histology preparation were cut into 3–5 mm thick slices and fixed in 10% formalin. Additional liver slices from the same liver lobe were either frozen on dry ice for biochemical analyses or snap-frozen in liquid nitrogen for gene expression investigations and DNA extraction and stored at −80 °C.

Analysis of TG was performed using a colorimetric TG assay kit (item no. 10010303, Cayman Chemical, Ann Arbor, MI, USA). The liver tissue was homogenized in NP40 Substitute Assay Reagent containing EDTA-free HALT protease inhibitor cocktail (Thermo Fisher Scientific, Roskilde, Denmark). The homogenates were centrifuged at 10,000× *g* for 10 min at 4 °C. The supernatant was diluted 5-fold in NP40 Substitute Assay Reagent and run in triplicates according to the manufacturer’s protocol in a blinded and randomized manner. Analysis of TC was performed using a fluorometric cholesterol assay kit (Item No. 10007640, Cayman Chemical, Ann Arbor, MI, USA). Following in-house validation of this method, the previously described liver homogenate supernatant was diluted 300-fold in cholesterol assay buffer and run in triplicate according to the manufacturer’s protocol in a blinded and randomized manner. For analysis of VitC, the liver samples were homogenized in cold Dulbecco’s PBS (D8537, Sigma-Aldrich, Darmstadt, Germany), stabilized in 10% MPA-EDTA, and centrifuged at 16,000× *g* for 1 min at 4 °C. The supernatant was stored at −80 °C until analysis on HPLC, as described previously [43].

### 2.5. Histology

Formalin-fixed liver tissue was embedded in paraffin, sliced into 2–4 µm thick sections, and stained with hematoxylin and eosin (H&E) or Picro Sirius Red (PSR). Histopathological scoring of MASLD was performed by an experienced pathologist in a blinded and randomized manner and according to the scoring descriptions provided by Kleiner et al. [44] adapted to the guinea pig model [21]. To ensure reliability and reproducibility, scoring was verified by calculating Cohen’s weighted kappa index, with a kappa score of >0.80 for all parameters except for inflammation, where a kappa score of >0.70 was accepted, as previously described [45]. Steatosis, inflammation, and ballooning were assessed on the H&E-stained slides, while fibrosis was scored on the PSR-stained slides. Steatosis grade and location were evaluated at ×5 or ×10 magnification across the entire slide and graded as 0 (<5%), 1 (>5–33%), 2 (>33–66%), or 3 (>66%). To assess lobular inflammation, six lobules across the liver section were examined at ×20 magnification, and the number of inflammatory foci (≥3 adjacent inflammatory cells) were counted and graded as 0 (none), 1 (1 focus per ×200 field), 2 (2–4 foci per ×200 field), or 3 (>4 foci per ×200 field). As a measure of very severe inflammation, the presence of large inflammatory foci—defined as aggregates of inflammatory cells visible at a low magnification—was assessed via yes/no scoring. Hepatocyte ballooning was evaluated at ×40 magnification and scored as 0 (none), 1 (few ballooning hepatocytes), or 2 (many ballooning hepatocytes). Overall the disease severity was assessed based on the MASLD/NAFLD activity score (NAS), which is the sum of steatosis, lobular inflammation, and ballooning scores, ranging from 0–8 and categorized as no MASLD (0–2), borderline (3–4), and MASH (≥5) [44].

Portal inflammation scoring was adapted from Kleiner et al. [44] and the NASH clinical research network [46]. It was scored by assessing six portal areas at high magnification. The number of inflammatory foci (≥5 adjacent inflammatory cells) were counted and scored as 0 (none, <2 foci in total), 1 (mild, 2–3 foci in total), or 2 (more than mild, >3 foci in total). Portal inflammation was initially not assessed in the majority of the adult guinea pigs. In order to compare portal inflammation between the two models, both juvenile and adult H&E stains were randomized and blinded together, and portal inflammation was subsequently scored overall.

Fibrosis was scored as F0 (not present), F1 (A: mild perisinosoidal, B: moderate perisinosoidal, and C: portal/periportal), F2 (perisinosoidal and portal/periportal), F3 (bridging from central vein to central vein, from central vein to portal vein, and/or from portal vein to portal vein), and F4 (A: mild cirrhosis, B: moderate cirrhosis, and C: severe cirrhosis) [47]. The presence of portal fibrosis was assessed via yes/no scoring. As previously described, fibrosis was quantified as the relative area of collagen-stained tissue in relation to the total area of liver tissue using the Visiopharm software (version 2020.08.4.9377, VisioPharm, Hørsholm, Denmark) [45].

### 2.6. Gene Expression

The RNA extraction, reverse transcription, and quantitative polymerase chain reaction (qPCR) protocols were performed as previously described [48]. Briefly, 50 mg of liver tissue from each animal was homogenized in 1 mL MagMax Lysis/Binding Solution Concentrate (Thermo Fisher, Waltham, MA, USA) containing 0.7% β-mercaptoethanol (Sigma Aldrich, St. Louis, MO, USA) and centrifuged at 10,000 RPM for 1 min at 4 °C. The supernatant was collected and stored at −20 °C for 24 h. Subsequently, RNA isolation was carried out using the MagMax-96 Total RNA Isolation Kit (Thermo Fisher, Waltham, MA, USA). For cDNA synthesis, 500 ng of RNA underwent reverse transcription (High Capacity cDNA Reverse Transcription Kit; Thermo Fisher, Waltham, MA, USA) using a 2720 Thermal Cycler (Applied Biosystems, Foster City, CA, USA) with the following conditions: 25 °C for 10 min, 37 °C for 120 min, and 85 °C for 5 s. Before proceeding with qPCR analysis of target genes, all cDNA samples were verified to be free of genomic DNA contamination using an intron-spanning primer set (β-actin, Table 1) [49].

For qPCR analysis, 2 µL of 3 ng/µL cDNA was added to 8 µL Master Mix (5 µL PowerUp SYBR Green Master Mix, 1 µL Primer mix, and 2 µL RNAse-free water). Triplicate samples were analyzed using the StepOnePlusTM Real Time PCR system (Applied Biosystems, Foster City, CA, USA) with the following conditions: 50 °C for 2 min, 95 °C for 5 min, followed by 40 cycles of 95 °C for 10 s, 60 °C for 10 s, and 72 °C for 20 s. Melting curves were examined for each primer set to validate product specificity. Primer sequences were designed using Primer3 or obtained from previous publications [48,49,50,51] (Table 1). Newly designed primers were sequenced (Eurofins, Ebersberg, Germany), and their target specificity was confirmed (NCBI BLAST). Dynactin subunit 5 (*DCTN5*) showed similar expression between all groups and was used as the reference gene [50]. qPCR was run on a subset of animals from each group (n = 7 for LFHC and HFLC; n = 8 for LFLC and HFHC). To ensure gene expression representative of the whole group, a subset of animals was selected via block-randomization based on histological inflammation scores.

**Table 1:** Primer sequences used for gene expression.

Gene	Accession No.	Forward	Reverse	Product (bp)
*TNF* (TNFα) [51]	NM_001173025.1	GCCGTCTCCTACCCGGAAAA	TAGATCTGCCCGGAATCGGC	203
*NLRP3*	XM_003479423.4	AAGTGGGGACCCATAAGGAC	GTAGCCAGCCAGCTTACACC	108
*CXCL8* (IL8)	NM_001173399.2	GGCAGCCTTCCTGCTCTCT	CAGCTCCGAGACCAACTTTGT	67
*IL18*	AB025722.1	CTCCGACTGTGCAGACAATG	TACACCTCTCGCGTTGCTAT	76
*CCL2* (MCP1) [48]	NM_001172926.1	TGCCAAACTGGACCAGAGAA	CGAATGTTCAAAGGCTTTGAAGT	75
*SOD1* [49]	U39844	TGTCCATGAGTTTGGCGATA	ATTTGCTCCGGAGAGTGAGA	193
*TET1*	XM_013156854.2	CAGTCAACGGCAACCTGAAA	ACCACTGTAATTCCGCCTGA	235
*TET2*	XM_003468037.3	AACTTCTGCGACTTCCAGGA	GGGTAAGAGCTGACTGGGTT	205
*TET3*	XM_013151565.2	GTGATGCCTCTTGTCCTCCT	GAGAGGCAAGAAGAGGGGTT	239
*ACTB* (β-ACTIN) [49]	AF508792	GTAAGGACCTCTATGCCAACACA	ATGCCAATCTCATCTCGTTTTCT	346
*DCTN5* [50]	XM_003477819.4	TTGACGGGATTCTGAGGTGC	CACAACACTGACTGGCGACT	122

Previously published primer pairs are referenced in square brackets. TNFα: tumor necrosis factor α; NLRP3: nucleotide-binding domain leucine-rich—containing family pyrin domain—containing-3; IL8: interleukin 8; IL18: interleukin 18; MCP1, monocyte chemotactic protein 1; SOD1: superoxide dismutase 1, TET1, TET2, TET3: ten–elven translocation methylcytosine dioxygenase 1-3; β-ACTIN: beta actin; DCTN5, dynactin subunit 5.

### 2.7. Global Methylation and Hydroxymethylation Levels

Global methylation and hydroxymethylation levels were analyzed using mass spectrometry as previously described [52]. In short, DNA was extracted from 20–25 mg of liver tissue using the DNeasy Blood & Tissue mini kit (Qiagen, Kista, Sweden) according to the manufacturer’s instructions. RNA-free genomic DNA was obtained by incubating the samples for 2 min at room temperature with 4 µL of RNase A (100 mg/mL, Macherey-Nagel GmbH & Co., Düren, Germany). Column washes were performed according to the kit protocol to remove digested RNA, and the DNA was eluted in water and assessed for concentration (*A*_260_) using a NanoDrop ND1000 instrument (Thermo Fisher, Waltham, MA, USA). Following extraction, DNA hydrolysis was performed using nucleoside digestion mix (New England BioLabs, Ipswich, MA, USA). Briefly, 0.5 µg of DNA was mixed with 2 µL of reaction buffer, 1 µL of nucleoside digestion mix, and water to a final volume of 20 µL.

The samples were desalted using a homemade filter composed of a plug of C18 filter pad (Empore) stuffed into a 200 µL pipet tip that was loaded with 60 µL (~1 µg) of poly-graphitic carbon resin (PGC, HyperCarb, Thermo Scientific, San Jose, CA, USA). The filter was washed with 100 µL of 0.1% trifluoroacetic acid (TFA) prior to use. The digested DNA samples were mixed with 100 µL of 0.1% TFA (pH = 2~3), loaded onto the filter, and washed once with 100 µL of 0.1% TFA. Nucleosides were eluted with 60 µL of a buffer composed as 60% acetonitrile (ACN) and 0.1% formic acid.

Nano liquid chromatography (nLC) was configured with a two-column system consisting of a 300 µm ID × 0.5 cm C18 trap column (Dionex) and a 75 µm ID × 25 cm Reprosil-Pur C18-AQ (3 µm; Dr. Maisch GmbH, Ammerbuch-Entringen, Germany) analytical nano-column packed in-house using a Dionex RSLC Ultimate 3000 (Thermo Scientific, San Jose, CA, USA). nLC was coupled online to an Orbitrap Fusion Lumos mass spectrometer (Thermo Scientific, San Jose, CA, USA). The spray voltage was set to 2.3 kV, and the temperature of the heated capillary was set to 275 °C. The full scan range was 100−800 *m*/*z*, acquired in the Orbitrap at a resolution of 120,000. Quantification was performed by manually extracting the area under the extracted ion chromatogram using the software Xcalibur version 4.1. The extracted signals were the protonated nucleobases cytosine (112.0505), methylcytosine (126.0662), and hydroxymethylcytosine (142.0611).

### 2.8. Statistics

Statistical analyses were conducted using GraphPad version 9.4.1 (GraphPad Prism software, La Jolla, CA, USA). For continuous data with a Gaussian distribution and equal variances between groups, Student’s *t*-test or two- or three-way ANOVA were applied. A repeated measures ANOVA was applied for body weights. These results are presented as means with standard deviations (SDs). In cases where the data did not adhere to a Gaussian distribution, transformation was applied followed by re-analysis, reported as medians with 25th and 75th percentiles. For the remaining data or in the case of ordinal data, non-parametric analysis was performed using either a Kruskal–Wallis’ test and Dunn’s test for multiple comparisons or a Mann–Whitney test. The results are presented as medians with 25th and 75th percentiles. Dichotomous data were analyzed busingFisher’s exact test and presented as percentages.

The gene expression data were analyzed using the ∆∆CT method and presented as log2-fold changes with ranges [53]. A two-way ANOVA was used for the analysis of ∆∆CT values and their standard deviations for superoxide dismutase 1 (*SOD1*), *TET1*, *TET2*, and *TET3*. To assess the overall effects of inflammatory gene expression patterns, a three-way ANOVA of all inflammatory genes’ ∆∆CT values and standard deviations was performed.

## 3. Results

### 3.1. Body Weights and Energy Intake

**Figure 2:**
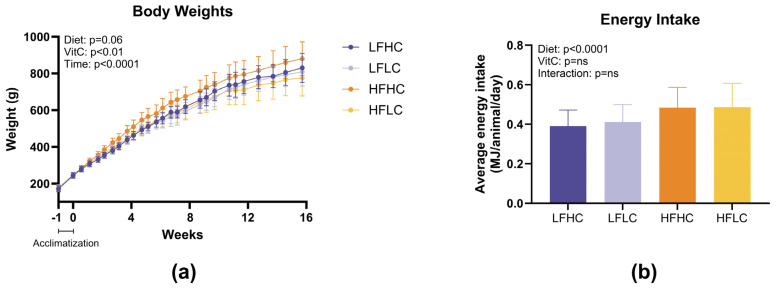
Body weights and energy intake. (**a**) Body weights across the experimental period, where week −1 to 0 was the week of acclimatization following arrival. Data were analyzed using a 3-way ANOVA with repeated measures. The overall effects of diet, vitamin C (VitC), and time are shown on the graph. Data are presented as means with SDs. (**b**) Average energy intake across the experimental period estimated pr. animal from the group feed intake was analyzed with a 2-way ANOVA and presented as means with SD. LFHC: low-fat high-VitC, LFLC: low-fat low-VitC, HFHC: high-fat high-VitC, HFLC: high-fat low-VitC. ns: not statistically significant; SD: standard deviation.

To establish a guinea pig model of pediatric MASH and study the contribution of VitC on the development of the disease, we subjected 1–2 weeks old guinea pigs for 16 weeks to diet either low or high in fat and carbohydrates, supplemented or not with vitamin (Figure 2). Both low VitC groups exhibited lower weights compared to their high VitC counterparts (*p* = 0.006) (endpoint weights: LFHC 823 g ± 80 g; LFLC 809 g ± 77 g; HFHC 872 g ± 94 g; HFLC 767 g ± 92 g) (Figure 2a). This effects of VitC cannot be attributed to variations in feed intake, as both HF groups showed similar energy intakes (gram feed intake) throughout the experimental period, as did the two LF groups (Figure 2b). The effects of high-fat, high-carbohydrate intake on body weights did not reach statistical significance (*p* = 0.060) (Figure 2a).

### 3.2. Plasma and Liver Biochemistry

As a measure of liver health, plasma levels of ALT, AST, and GGT were assessed (Table 2). ALT and GGT were increased in both high-fat groups (*p* = 0.001 and *p* < 0.0001, respectively), supporting liver stress in these groups. AST values were not different between groups. The plasma lipid profile was evaluated via TG and TC (Table 2), with the LC diet leading to a significantly higher plasma TG (*p* = 0.004). In the low-fat groups, TC was below the detection level of 0.65 mM. Thus, TC levels could only be compared between the HF groups, showing no significant difference. As expected, the VitC was lower in the LC groups compared to the HC groups (*p* < 0.0001) (Table 2).

Liver TG and TC levels were higher in the high-fat diet compared to the low-fat diet groups (*p* < 0.0001) (Table 2). Reflecting plasma concentration, total hepatic VitC was lower in the LC groups than in the HC groups (*p* < 0.0001) (Table 2). Of note, there was also an effect of HF diet alone (*p* = 0.004), where VitC was lower in HF compared to LF. There was, however, no interaction effect, i.e., a lower VitC amount in the HF, compared to LF when animals were deprived of VitC.

### 3.3. Juvenile Histopathology

**Figure 3:**
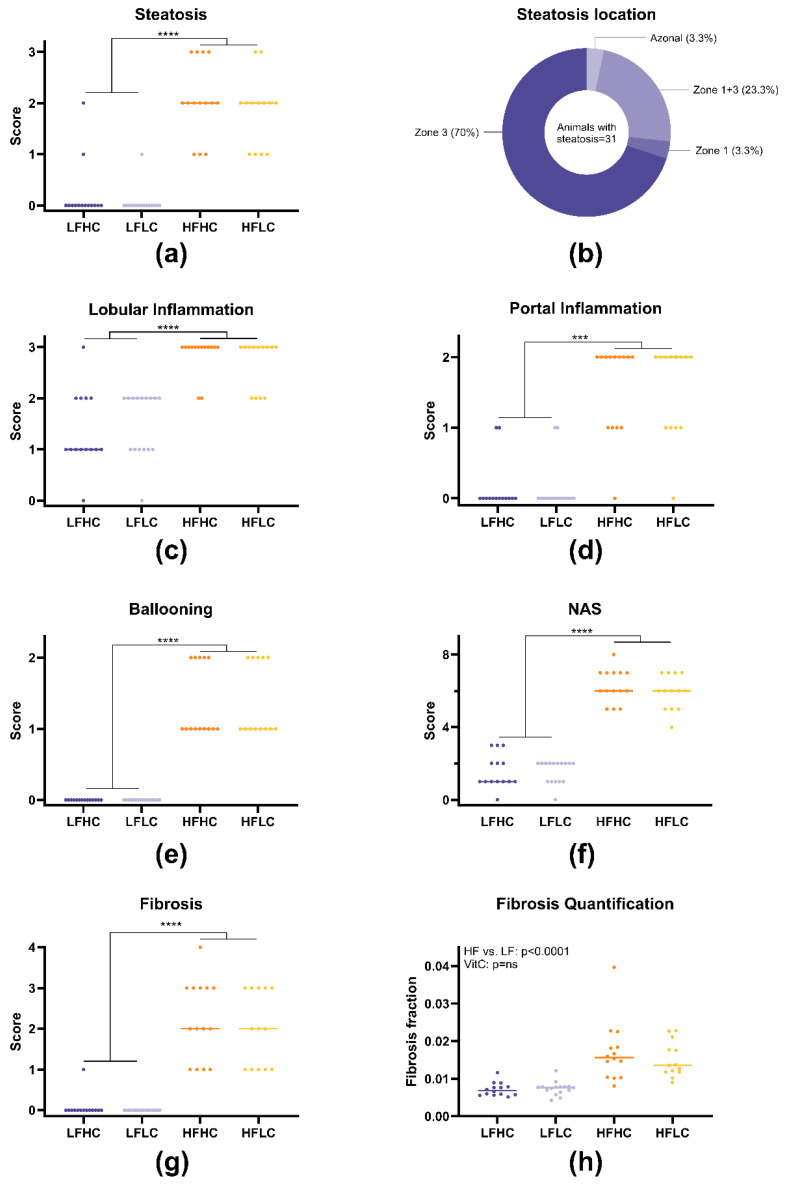
Histopathological scoring and fibrosis quantification. All data except steatosis location are presented as individual scores with medians. (**a**) Steatosis scores on a scale of 0–3, (**b**) prevalence of different steatosis locations, (**c**) lobular inflammation scores on a scale of 0–3, (**d**) portal inflammation scores on a scale of 0–2, (**e**) ballooning scores on a scale of 0–2, (**f**) MASLD/NAFLD activity scores (NAS) on a scale of 0–8, (**g**) fibrosis scores on a scale of 0–4, (**h**) fibrosis fractions. Histopathological scoring data were analyzed with non-parametric Kruskal–Wallis’ test and Dunn’s test for multiple comparisons. Fibrosis fractions were log-transformed and analyzed via two-way ANOVA. *** *p* < 0.001, **** *p* < 0.0001, ns: not statistically significant. LFHC: low-fat high-VitC, LFLC: low-fat low-VitC, HFHC: high-fat high-VitC, HFLC: high-fat low-VitC.

**Figure 4:**
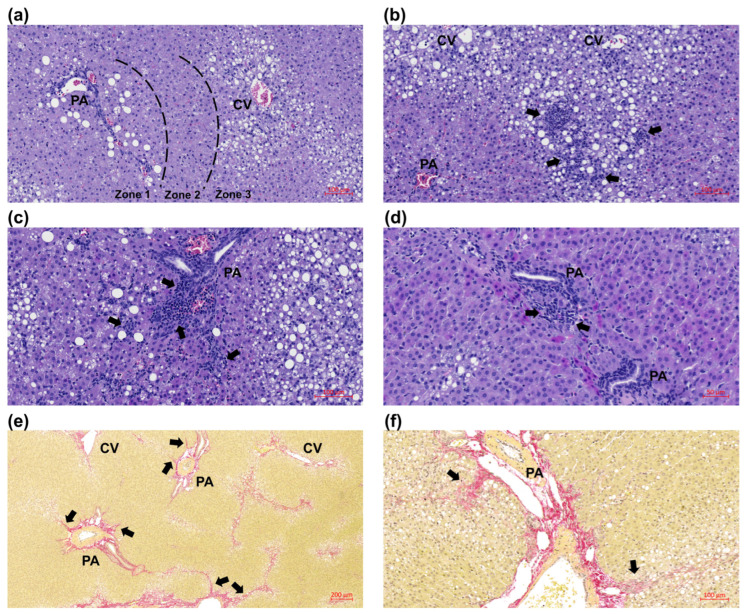
Representative examples of hepatic histology. (**a**) Zone 1 + 3 steatosis with a clear separation of zones 1 and 3, (**b**) severe lobular inflammation (arrows), (**c**,**d**) portal inflammation (arrows), (**e**,**f**) expanding portal fibrosis (arrows). CV: central vein, PA: portal area.

The HF diet induced histopathological changes compatible with human MASH in all of the juvenile guinea pigs (Figure 3 and Figure 4). All of the HF-fed guinea pigs developed steatosis, with 21/31 (70%) having steatosis located in zone 3. Interestingly, 7/31 (23%) displayed steatosis in both zones 1 and 3, with a clear separation of the zones (Figure 3b and Figure 4a). The HF diet induced severe lobular inflammation (Figure 3c and Figure 4b), and 25/27 (93%) in the HF groups displayed portal inflammation (Figure 3d and Figure 4c,d). Based on a NAS score of ≥5 as the threshold criterion for MASH, 26/27 (96%) in the HF groups had definite MASH, with a median NAS score of 6 in both HF groups (Figure 3f). Furthermore, the HF groups displayed advanced fibrosis, with a median score of 2 (Figure 3g), supported by fibrosis quantification (Figure 3h). In 16/27 (59%), fibrosis was present in the portal areas (Figure 4e,f). Low VitC did not induce significant histological differences in the LF nor HF groups; however, the median score for lobular inflammation in the LFLC group was numerically higher than in the LFHC group, though not reaching statistical significance. Furthermore, in the HFLC group, 9/13 (69%) displayed large inflammatory foci (Figure 4b), while this was only the case for 5/14 (36%) in the HFHC group (*p* = 0.128, Fisher’s exact test).

### 3.4. Juvenile Compared to Adult Histopathology

To determine if our juvenile guinea pig model differentiates from the guinea pig model of adult MASH [21,22,45,48,54,55], the histopathological data from the two HF groups were compared to histological data from previously published studies of adult female guinea pigs (groups: HFH and HFL [45], HF [55], HFD pre-intervention [54]). The adult guinea pigs were from the same breeder, fed the same HF diet for the same duration (16 weeks), and kept in the same facilities and under comparable conditions as in the current study of juvenile guinea pigs.

**Figure 5:**
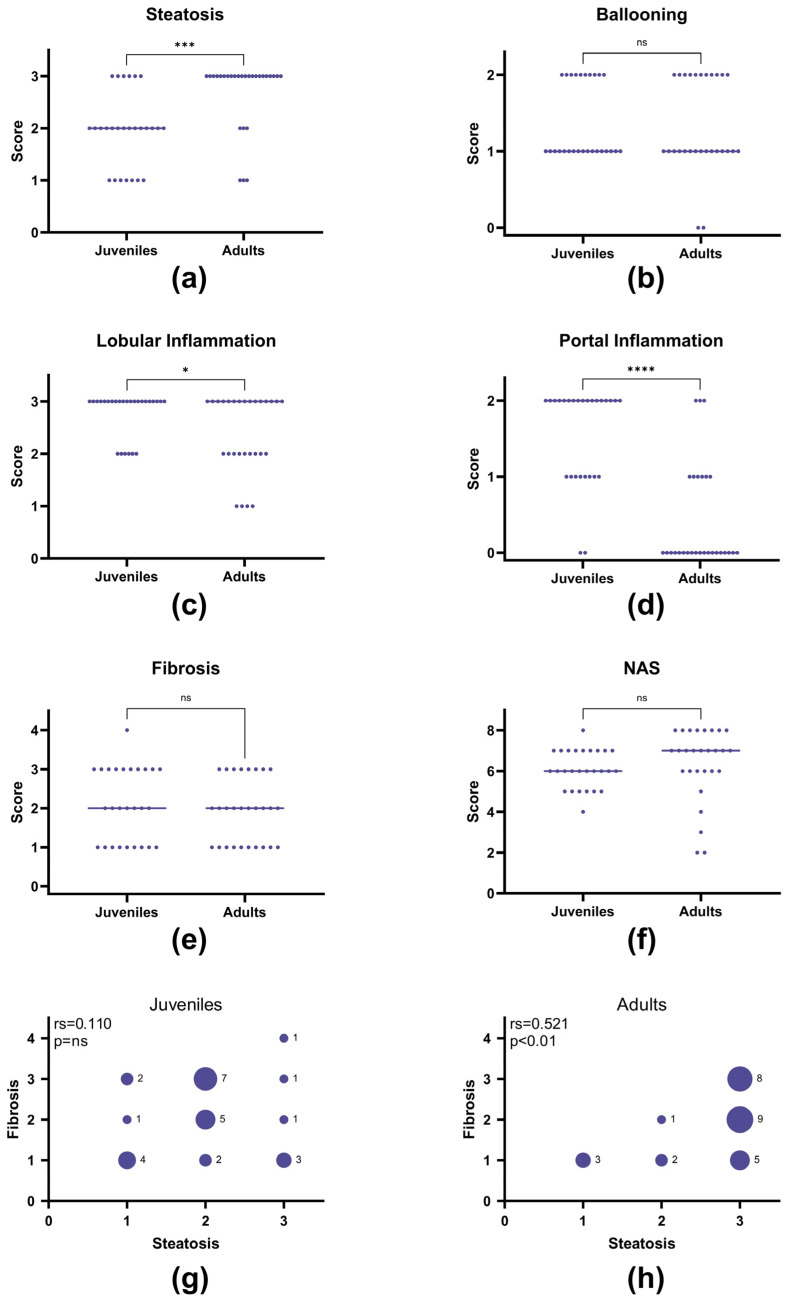
Histopathological comparisons of juvenile (n = 27) and adult (n = 28, historical data [38,46,47]) guinea pigs. All guinea pigs were kept on a high-fat diet for 16 weeks, originated from the same breeder, and kept in the same facilities under the same conditions. (**a**) Steatosis scores on a scale of 0–3, (**b**) ballooning hepatocytes on a scale of 0–2, (**c**) lobular inflammation scores on a scale of 0–3, (**d**) portal inflammation scores on a scale of 0–2, (**e**) fibrosis scores on a scale of 0–4, (**f**) MASLD/NAFLD activity scores (NAS) on a scale of 0–8, (**g**) individual fibrosis and steatosis scores of juvenile guinea pigs plotted against each other, (**h**) individual fibrosis and steatosis scores of adult guinea pigs plotted against each other. The histopathological scores (**a**–**f**) were analyzed with a Mann–Whitney test and presented as individual values with medians. The steatosis vs. fibrosis scores (**g**,**h**) were analyzed with Spearman’s correlation, with the correlation coefficient (rs) and *p*-value presented on each graph. * *p* < 0.05, *** *p* < 0.001, **** *p* < 0.0001, ns: not statistically significant.

Hepatic histopathology showed that the juvenile guinea pigs had less steatosis than the adults (*p* = 0.0002) (Figure 5a). Most interestingly, the juvenile guinea pigs displayed increased lobular and portal inflammation compared to the adults (*p* = 0.046 and *p* < 0.0001, respectively) (Figure 5c,d). Ballooning, fibrosis, and NAS scores were similar between the age groups (Figure 5b,e,f). In the adult model, the steatosis score correlated with the fibrosis score (*p* = 0.005), and severe fibrosis was only present together with severe steatosis (Figure 5h). This was not the case in the juvenile guinea pigs, where no correlation was present, and severe fibrosis combined with only mild steatosis was found in some animals (Figure 5g). Collectively, this indicates a different and more severe disease development in the juvenile MASH guinea pigs and validates our model for the study of pediatric MASH.

### 3.5. Gene Expression and Epigenetic Modifications

**Figure 6:**
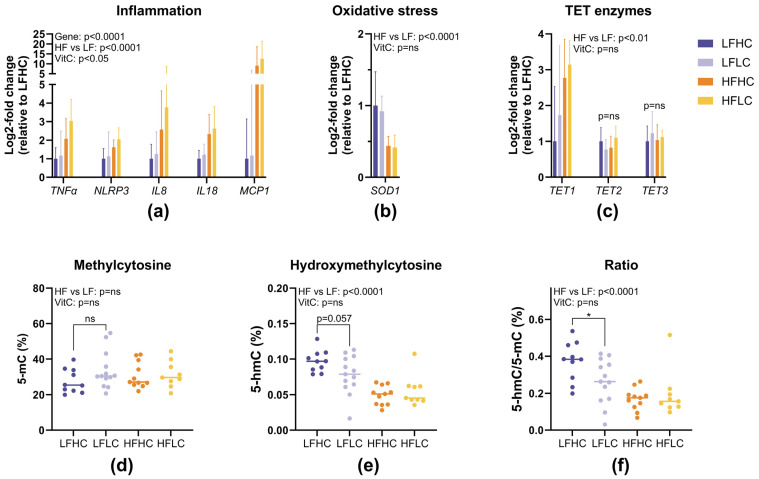
Gene expression and global methylation and hydroxymethylation levels from liver tissue. Gene expression data (**a**–**c**) are presented as means with ranges expressed as log2-fold changes relative to LFHC. Gene expression was studied on a subset of animals from each group, with n = 7 for LFHC and HFLC and n = 8 for LFLC and HFHC. The subset of animals was selected randomly via block-randomization based on histological lobular inflammation score to ensure gene expression representative for the whole group (**a**) Expression of inflammatory genes. All inflammatory genes were analyzed via three-way ANOVA. (**b**) Expression of SOD1 as a marker of oxidative stress, analyzed via two-way ANOVA. (**c**) Expression of TET enzymes, analyzed via two-way ANOVA. Global methylation and hydroxymethylation levels (**d**–**f**) were studied on a subset of animals from each group (LFHC: n = 10; LFLC: n = 13; HFHC: n = 11; HFLC: n = 9), presented as individual values with the median and analyzed via two-way ANOVA to assess the overall effects of diet and VitC. A *t*-test was used to compare the LF groups. (**d**) Global methylcytosine (5-mC) expressed as percent of total cytosine. (**e**) Global hydroxymethylcytosine (5-hmC) expressed as percent of total cytosine. (**f**) Ratio between hydroxymethylated cytosine and methylated cytosine expressed as percent. * *p* < 0.05. TNFα: tumor necrosis factor α, NLRP3: nucleotide-binding domain leucine-rich—containing family pyrin domain—containing-3, IL8: interleukin 8, IL18: interleukin 18, MCP1: monocyte chemotactic protein 1, SOD1: superoxide dismutase 1, TET1, TET2, TET3: ten–elven translocation methylcytosine dioxygenase 1-3, LFHC: low-fat high-VitC, LFLC: low-fat low-VitC, HFHC: high-fat high-VitC, HFLC: high-fat low-VitC. ns: not statistically significant.

Since the histopathological scores for lobular inflammation indicated that low VitC may increase liver inflammation, the expression of key inflammatory genes was investigated. As expected, the HF groups had higher expression of inflammatory genes when compared to the LF groups (*p* < 0.0001) (Figure 6a). The effects of an LC diet were also significant (*p* = 0.049) and were most pronounced in the HFLC animals across all of the tested inflammatory genes (Figure 6a). The HF groups showed lower expression of SOD1, a marker of oxidative stress, compared to the LF groups (*p* < 0.0001), while there was no effect of a LC diet (Figure 6b).

To investigate whether VitC status affected the expression of VitC-sensitive enzymes controlling hydroxymethylation, the gene expression patterns of the three TET enzymes were examined (Figure 6c). While *TET2* and *TET3* did not show any differences in expression patterns between the groups, *TET1* expression showed the same expression pattern as the inflammatory genes, with a higher expression in the HF groups and with the highest expression in HFLC (*p* = 0.002). However, depletion of VitC did not have a detectable effect on *TET1* expression.

To study diet-induced changes oin the epigenome and as an indirect measure of TET enzyme activity, the global levels of methylcytosine and hydroxymethylcytosine were analyzed (Figure 6d–f). No differences in global methylcytosine levels were detected (Figure 6d), but the HF groups had lower levels of global hydroxymethylcytosine compared to the LF groups (*p* < 0.0001) (Figure 6e). This difference persisted when comparing the ratio of hydroxymethylcytosine to methylcytosine (*p* < 0.0001) (Figure 6f). To isolate the effect of VitC from the overpowering effect of the HF diet, LFHC was compared to LFLC. There was no difference in global methylcytosine or hydroxymethylcytosine levels (*p* = ns and *p* = 0.057, respectively), but the ratio of hydroxymethylcytosine to methylcytosine was lower in LFLC compared to LFHC (*p* = 0.034).

## 4. Discussion

Here, we provide evidence that guinea pigs could serve as a model of pediatric MASH, where a diet rich in fat and carbohydrates induced plasma and liver biochemistry as well as hepatic histopathological hallmarks of pediatric MASH. Our findings further confirm that juvenile/pediatric MASH histopathology is a distinct disease phenotype compared to adult MASH. Moreover, we showed that low VitC intake increased the hepatic expression of inflammatory genes, indicating a possible role of VitC in disease severity.

Portal pathology in this guinea pig model of pediatric MASH aligned closely with histopathological findings in children and adolescents. Although human studies consistently indicate that portal fibrosis and inflammation are more prevalent in pediatric cases compared to adults, the distribution between the different types varies across studies. Schwimmer et al. [7] introduced the classification of pediatric MASH into types I and II and found that 51% displayed type II MASH, while 16% showed an overlap between the types. Subsequent investigations, however, reported a lower incidence of distinct type II MASH (8–28%). Instead, a majority of children and adolescents exhibited overlapping pathology (50–82%) [9,10,11,12]. Although portal/zone 1 injury (fibrosis and/or inflammation) is more prevalent in children and adolescents compared to adults, it typically coexists with zone 3 (lobular) injury rather than presenting in isolation [6,10]. In the current guinea pig model of pediatric MASH, both portal and lobular inflammation and fibrosis occurred concurrently, reflecting the overlap between type I and II pathology reported in human pediatric MASH. Consequently, this model not only captures hallmarks of pediatric MASH histopathology but also reflects the most prevalent histopathological type observed in clinical studies.

When compared to their adult counterparts, the juvenile guinea pigs developed less liver steatosis. This finding is most likely attributed to an intense growth rate experienced by the juvenile guinea pigs, leading to increased energy utilization and a reduction in caloric surplus. Steatosis is recognized as a key driver of MASH [56], and the reduced steatosis could suggest a less severe disease in the juvenile guinea pigs. However, despite the seemingly milder lipid load, the animals developed an equivalent amount of fibrosis to that observed in the adult guinea pigs and displayed more lobular and portal inflammation. These findings indicate that the juvenile guinea pigs experienced comparable or even greater liver injury than the adults despite a milder steatotic condition. This observation aligns with the more aggressive disease development seen in children and adolescents [5,6]. Furthermore, the intense portal inflammation in the juvenile guinea pigs compared to the adults underlines a distinct histopathological difference between the two models. Collectively, these characteristics show that juvenile guinea pigs represent a highly relevant pre-clinical model of human pediatric MASH.

The Kleiner scoring system for lobular inflammation stops at a maximal score of three [44]. Due to the semi-quantitative nature of this scoring scheme, it is therefore not possible to distinguish between severe and very severe inflammation. The animals in the LFLC group showed a numerically higher median score of lobular inflammation compared to LFHC, possibly indicating an increased inflammatory condition due to low VitC, though this was not found to be statistically significant and therefore remains speculative. Very severe inflammation was scored as the presence of large inflammatory foci and noted in more than two-thirds (69%) of the HFLC group and in 36% of the HFHC guinea pigs. Subsequent quantification of the hepatic expression of inflammatory target genes supported increased mRNA expression in the low-VitC groups. This finding aligns with previous observations showing upregulation of inflammatory pathways in guinea pigs with MASH when switched to a LFLC diet compared to those switched to a LFHC diet [45]. MASH is essentially an inflammatory condition, partly driven by the generation of reactive oxygen species (ROS) during hepatic oxidation of fatty acids [57]. Antioxidants such as VitC play a crucial role in neutralizing ROS and preventing the oxidative stress they induce. Thus, a low-VitC status may exacerbate oxidative stress, leading to the activation of inflammatory pathways like the NLRP3 inflammasome and the release of inflammatory cytokines and chemokines such as TNFα, IL8, IL18, and MCP1 that recruit inflammatory cells and activate hepatic stellate cells [58,59,60].

Aligning with previous studies, the current study showed that guinea pigs subjected to a HF diet displayed lower hepatic VitC levels compared to the LF groups [24,45]. This may suggest an increased utilization of antioxidants due to increased ROS in the HF groups, consequently exhausting antioxidant reserves as lipid stress persists. A state of increased oxidative stress in the HF groups is substantiated by lower *SOD1* expression in the HF groups. Superoxide dismutase (SOD) is an important cellular defense against ROS [61], and when the capacity of the mitochondrial β-oxidation is surpassed due to the high lipid load, electrons start to “leak” from the electron transport chain, leading to overproduction of ROS and stressed mitochondria [62]. As also seen in human MASH, this leads to a depletion of SOD capacity, further exacerbating oxidative stress [63]. *SOD1* expression positively correlates with SOD protein levels in guinea pigs [49], and aligning with the current study, low SOD levels have previously been reported in guinea pigs with MASH [21], confirming the state of hepatic oxidative stress. In adult humans, obese individuals have lower serum VitC concentrations compared to lean individuals despite similar daily intake, thus suggesting additional supplementation in obese individuals [26,64]. In addition to an increased turnover of antioxidant capacity due to ROS, it could also be speculated that reduced absorption due to the composition of a HF diet could contribute to reduced bioavailability of ingested VitC, in turn exacerbating a low antioxidant status. Recent data from NHANES (2012–2020) report a negative correlation between the composite dietary antioxidant index and hepatic fibrosis (liver stiffness) in MASLD, linking antioxidant intake to reduced disease incidence and severity [65]. As reduced VitC intake in children has been linked to increased ballooning hepatocytes [34], our findings highlight the importance of further study of the role of VitC intake to improve liver health in children affected by MASLD and MASH.

Another role of VitC is as a co-factor in TET enzyme activity [36,66]. By contributing to the conversion of methylated cytosines to hydroxymethylated cytosines, TET enzymes participate in the epigenetic regulation of gene expression. If their activity is compromised, the epigenetic machinery is essentially compromised, impeding the cell’s ability to respond appropriately to stress during MASH development. In the current study, the observed effects on *TET* expression were subtle, with no differences in *TET2* and *TET3*, while the HF diet increased the expression of *TET1*. There was no significant effect of low VitC on *TET1* expression—perhaps obscured by large variation—but the pattern of *TET1* expression appeared to be higher in LC groups, mirroring that of the inflammatory genes. This may reflect the exacerbated disease that low VitC status appears to induce, possibly indicating a link between *TET1* expression and inflammatory gene regulation. It may also be a compensatory response to increase the transcription of *TET1* if low-VitC status decreases the activity of the TET1 enzyme, although this remains speculative, as there are currently no validated assays to directly assess TET activity. As an indirect measure of TET activity, the ratio of global hydroxymethylated cytosine to methylated cytosine was lower in LFLC compared to LFHC. This reduction may be attributed to decreased TET activity mediated by low VitC levels. Interestingly, these findings show that a low VitC status alters the epigenome, and the implications of this need to be further explored. There was no effect of diet on the global methylation levels, aligning with the inconsistent findings in the literature regarding the link between MASLD and global hepatic methylation patterns [67,68]. However, the HF diet induced lower global hydroxymethylation levels, where previous studies primarily found locus-specific changes in hydroxymethylation and not changes in global hydroxymethylation levels [69,70].

Whereas the adult MASH guinea pig model utilizes female animals to avoid hierarchal stress, the current study was performed in male juveniles. By study termination at 16 weeks, aggressive behavior was evident, leading to preventive isolation of some animals and confirming that male guinea pigs are not suitable for the adult model. Consequently, when comparing the histopathology of the current juvenile guinea pigs to previously collected data from adult guinea pigs, sex may be a confounding factor. In humans, males are more susceptible to developing MASH than females [71]; a trend also observed in children and adolescents, although it varies depending on the study [2]. Similarly, estrogen seems to protect mice from MASH and insulin resistance [72]. However, a juvenile murine MASH model of both sexes revealed comparable liver damage between male and female mice, thus not supporting sex-associated differences in disease development and severity juvenile mice [18]. Currently, studies comparing sex-associated differences in MASH development in guinea pigs are lacking, but adult female guinea pigs develop definite MASH as well as glucose intolerance within 16 weeks on a HF diet, diminishing any potential estrogen-protective effects on MASH in this species [45,50]. Another aspect of the guinea pig model is the specific requirements in this species compared to other rodents such as mice and rats. Apart from a dependency on dietary VitC intake like humans (and contrary to almost all other mammals), guinea pigs are obligatory vegetarians and depend on intestinal fermentation for the digestion of plant material. Hay is thus a mandatory part of the feeding regime to ensure a functional gut microbiota. In addition, the size of the animal—reaching a body weight of more than 750 g at the termination of the current study—increases housing and management requirements such as cage space and feed amount. For intervention studies, e.g., testing drug interventions, the amount of compound necessary will likely also exceed that of studies performed in the smaller rodents. Importantly, while this may increase costs and potentially limit the use of the model, the size of guinea pigs allows us to obtain larger samples and monitor multiple parameters both during the concourse of a study and following termination. In turn, this diminishes the need for interim terminations, ultimately reducing the number of experimental animals. Reviewing the present findings, the recorded similarity with the hepatic pediatric MASH phenotype and the dependency on a dietary VitC supply outweighs the above-outlined limitations and strongly supports this animal model as unique for further investigations of the mechanisms of pediatric MASH and the potential combined effects of VitC in this disease.

## 5. Conclusions

In conclusion, our study indicated that guinea pigs constitute a relevant and novel model of pediatric MASH, showing comparable histopathological hallmarks to those found in children and adolescents. We showed that low VitC levels may exacerbate inflammation in juveniles, possibly through epigenetic regulation of gene expression. This supports a beneficial effect of VitC-rich food to be considered as part of dietary interventions for ameliorating liver health in children and adolescents affected by MASH. In this way, the current findings support micronutrients as part of preventive and therapeutic modalities, deserving further attention in future explorations.

## Figures and Tables

**Table 2:** Plasma and liver biochemistry.

Experimental Groups					2-Way ANOVA		
	LFHC	LFLC	HFHC	HFLC	HF vs. LF	VitC	Int.
Plasma							
ALT (U/L) ^1^	68 (56–87)	62 (51–74)	105 (69–118)	86 (59–94)	**	ns	ns
AST (U/L) ^1^	220 (86–472)	227 (89–382)	279 (157–390)	193 (147–372)	ns	ns	ns
GGT (U/L) ^1^	73 (64–80)	57 (49–72)	89 (73–105)	93 (77–113)	****	ns	*
TG (mM) ^2^	0.70 (0.60–0.81)	0.79 (0.62–1.03)	0.56 (0.53–0.69)	0.74 (0.65–1.00)	ns	**	ns
TC (mM) ^3^	<0.65	<0.65	6.51 (3.60–8.71)	8.88 (5.52–12.09)	-	ns	-
Total VitC (µM) ^1^	33.89 (26.91–38.77)	2.69 (2.27–2.88)	28.70 (21.59–38.39)	2.71 (2.49–2.88)	ns	****	ns
Liver							
TG (µmol/g) ^1^	16.3 (15.7–21.1)	19.9 (14.9–23.9)	36.8 (32.6–43.0)	38.7 (34.7–41.5)	****	ns	ns
TC (µmol/g) ^4^	17.7 ± 2.8	17.6 ± 4.4	39.7 ± 6.1	38.9 ± 6.6	****	ns	ns
TotalVitC (nmol/g) ^5^	1136 (886–1396)	68.0 (49.6–154)	889 (738–1036)	61.2 (33.3–107)	**	****	ns

Data are presented as medians with quartiles (Q25–Q75) in brackets or means ± SDs. ^1^ Log-transformed data were analyzed via two-way ANOVA. ^2^ Reciprocally transformed data were analyzed via two-way ANOVA. ^3^ Log-transformed data were analyzed via a Student’s *t*-test, TC data in the low-fat groups were under detection level of 0.65 mM. One animal from LFHC was excluded from the VitC analysis. ^4^ Data were analyzed via two-way ANOVA. ^5^ Square root-transformed data were analyzed via two-way ANOVA. * *p* < 0.05, ** *p* < 0.01, **** *p* < 0.0001. ALT: alanine aminotransferase, AST: aspartate aminotransferase, GGT: gamma-glutamyl transferase, TG: triglycerides, TC: total cholesterol, VitC: vitamin C, LFHC: low-fat high-VitC, LFLC: low-fat low-VitC, HFHC: high-fat high-VitC, HFLC: high-fat low-VitC, Int.: interaction.

## Data Availability

The raw data supporting the conclusions of this article will be made available by the authors on request.

## Supplementary Materials

The following supporting information can be downloaded at: https://www.mdpi.com/article/10.3390/nu17020291/s1, Table S1: Diet composition.
