# Correction: Pedersen et al. A Guinea Pig Model of Pediatric Metabolic Dysfunction-Associated Steatohepatitis: Poor Vitamin C Status May Advance Disease. *Nutrients* 2025, *17*, 291

**DOI:** 10.3390/nu18071067

**Published:** 2026-03-27

**Authors:** Kamilla Pedersen, Ankita Poojari, Simone Frederikke Colberg, Stine Marguerite Mechernsee, Jo Frøkjær Iversen, Romain Barrès, Jens Lykkesfeldt, Pernille Tveden-Nyborg

**Affiliations:** 1 Section of Preclinical Disease Biology, Department of Veterinary and Animal Sciences, Faculty of Health and Medical Sciences, University of Copenhagen, 1870 Frederiksberg, Denmark; kamilla.pedersen@sund.ku.dk (K.P.); jopl@sund.ku.dk (J.L.); 2 Thomas J. Long School of Pharmacy, University of the Pacific, Stockton, CA 95211, USA; apoojari@pacific.edu; 3 Novo Nordisk Foundation Center for Basic Metabolic Research, University of Copenhagen, 2200 Copenhagen, Denmark; jo.iversen@sund.ku.dk (J.F.I.); barres@sund.ku.dk (R.B.); 4 Institut de Pharmacologie Moléculaire et Cellulaire, CNRS and Université de Nice Côte d’Azur, 06560 Valbonne, France

## Error in Table

In the original publication [1], there was a mistake in Table 2 as published. Plasma total cholesterol (TC) values were mistakenly reported as log-transformed data. The corrected values are:

HFHC: 6.51 (3.60–8.71) [initially and faulty reported as 0.81 (0.56–0.94)]

HFLC: 8.88 (5.52–12.09) [initially and faulty reported as 0.95 (0.74–1.08)]

The corrected Table 2 appears below. The authors state that the scientific conclusions are unaffected. This correction was approved by the Academic Editor. The original publication has also been updated.

## Error in Figure and Legend

In the original publication, there was a mistake in the figure and legend for Figure 3. The figure and legend indicate fibrosis fraction as a percentage (%) and should only be fraction. The portal inflammation scale was misstated as 0–1 in the legend and should be corrected to 0–2. The correct Figure 3 and legend appear below. The authors state that the scientific conclusions are unaffected.

## Figures and Tables

**Figure 3:**
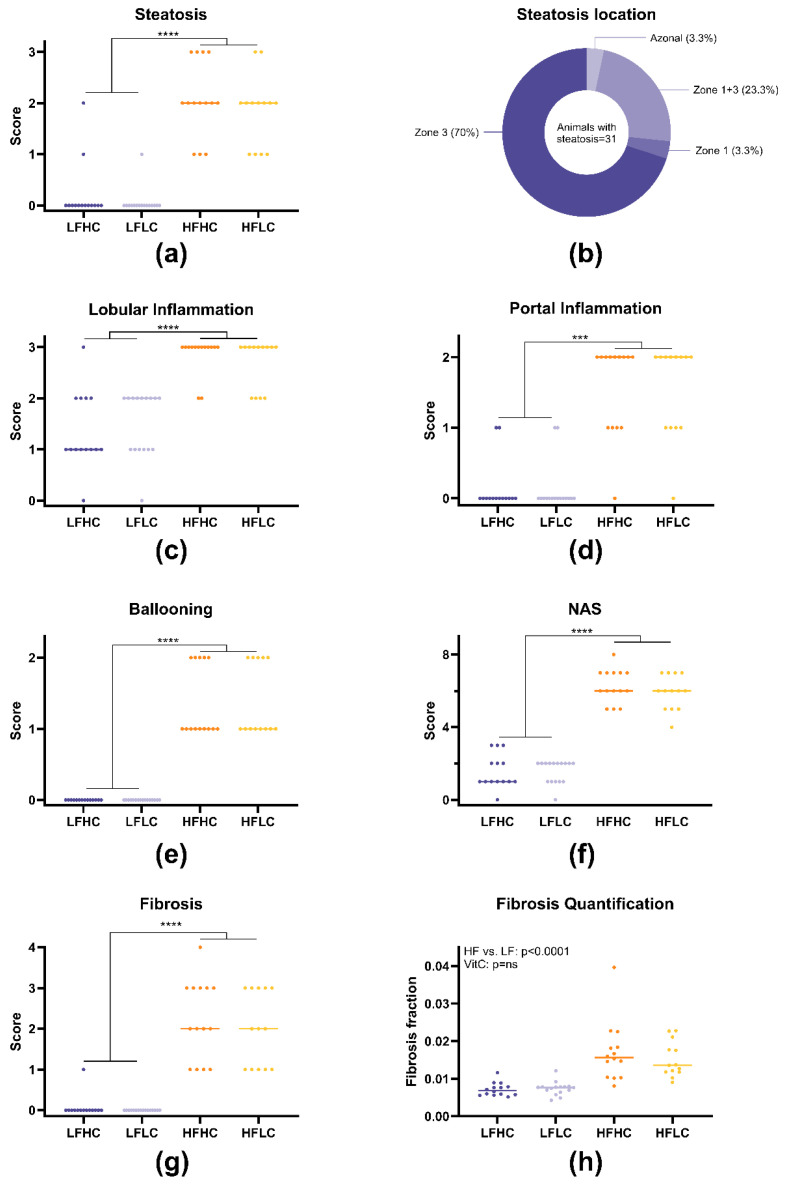
Histopathological scoring and fibrosis quantification. All data except steatosis location are presented as individual scores with medians. (**a**) Steatosis scores on a scale of 0–3, (**b**) prevalence of different steatosis locations, (**c**) lobular inflammation scores on a scale of 0–3, (**d**) portal inflammation scores on a scale of 0–2, (**e**) ballooning scores on a scale of 0–2, (**f**) MASLD/NAFLD activity scores (NAS) on a scale of 0–8, (**g**) fibrosis scores on a scale of 0–4, (**h**) fibrosis fractions. Histopathological scoring data were analyzed with non-parametric Kruskal–Wallis’ test and Dunn’s test for multiple comparisons. Fibrosis fractions were log-transformed and analyzed via two-way ANOVA. *** *p* < 0.001, **** *p* < 0.0001, ns: not statistically significant. LFHC: low-fat high-VitC, LFLC: low-fat low-VitC, HFHC: high-fat high-VitC, HFLC: high-fat low-VitC.

**Table 2:** Plasma and liver biochemistry.

Experimental Groups					2-Way ANOVA		
	LFHC	LFLC	HFHC	HFLC	HF vs. LF	VitC	Int.
Plasma							
ALT (U/L) ^1^	68 (56–87)	62 (51–74)	105 (69–118)	86 (59–94)	**	ns	ns
AST (U/L) ^1^	220 (86–472)	227 (89–382)	279 (157–390)	193 (147–372)	ns	ns	ns
GGT (U/L) ^1^	73 (64–80)	57 (49–72)	89 (73–105)	93 (77–113)	****	ns	*
TG (mM) ^2^	0.70 (0.60–0.81)	0.79 (0.62–1.03)	0.56 (0.53–0.69)	0.74 (0.65–1.00)	ns	**	ns
TC (mM) ^3^	<0.65	<0.65	6.51 (3.60–8.71)	8.88 (5.52–12.09)	-	ns	-
Total VitC (µM) ^1^	33.89 (26.91–38.77)	2.69 (2.27–2.88)	28.70 (21.59–38.39)	2.71 (2.49–2.88)	ns	****	ns
Liver							
TG (µmol/g) ^1^	16.3 (15.7–21.1)	19.9 (14.9–23.9)	36.8 (32.6–43.0)	38.7 (34.7–41.5)	****	ns	ns
TC (µmol/g) ^4^	17.7 ± 2.8	17.6 ± 4.4	39.7 ± 6.1	38.9 ± 6.6	****	ns	ns
TotalVitC (nmol/g) ^5^	1136 (886–1396)	68.0 (49.6–154)	889 (738–1036)	61.2 (33.3–107)	**	****	ns

Data are presented as medians with quartiles (Q25–Q75) in brackets or means ± SDs. ^1^ Log-transformed data were analyzed via two-way ANOVA. ^2^ Reciprocally transformed data were analyzed via two-way ANOVA. ^3^ Log-transformed data were analyzed via a Student’s *t*-test, TC data in the low-fat groups were under detection level of 0.65 mM. One animal from LFHC was excluded from the VitC analysis. ^4^ Data were analyzed via two-way ANOVA. ^5^ Square root-transformed data were analyzed via two-way ANOVA. * *p* < 0.05, ** *p* < 0.01, **** *p* < 0.0001. ALT: alanine aminotransferase, AST: aspartate aminotransferase, GGT: gamma-glutamyl transferase, TG: triglycerides, TC: total cholesterol, VitC: vitamin C, LFHC: low-fat high-VitC, LFLC: low-fat low-VitC, HFHC: high-fat high-VitC, HFLC: high-fat low-VitC, Int.: interaction.
