# Supplementary material for: A Guinea Pig Model of Pediatric Metabolic Dysfunction-Associated Steatohepatitis: Poor Vitamin C Status May Advance Disease
Source: Nutrients. 2025 Jan 15;17(2):291. doi: 10.3390/nu17020291 (PMC11767659; doi:10.3390/nu17020291)
Supplement: Supplementary file 1 [file nutrients-17-00291-s001.zip › nutrients-3398495-supplementary.pdf]

Supplementary Table S1. Diet composition

Product		LF 1740 mg/kg VitC	LF 156 mg/kg VitC	LF 0 mg/kg VitC	HF 1830 mg/kg VitC	HF 143 mg/kg VitC	HF 0 mg/kg VitC
**Alfalfa**	%	33	33	33	22	22	22
**Wheat**	%	3.86	4.41	4.44	10	10	10
**Barley**	%	10	10	10	-	-	-
**Wheat Bran**	%	15.55	15.55	15.55	-	-	-
**Sucrose**	%	-	-	-	14.83	15.41	15.38
**Inulin**	%	3	3	3	-	-	-
**Cellulose (Lignocellulose)**	%	4	4	4	4.6	4.6	4.6
**Sunflower meal**	%	5	5	5	3	3	3
**Soybean meal**	%	10.2	10.2	10.2	6	6	6
**Soybeans (full fat)**	%	1	1	1	2.6	2.6	2.6
**Soybean concentrate**	%	-	-	-	12	12	12
**Corn gluten feed**	%	8	8	8	-	-	-
**Amino acids**	%	0.65	0.65	0.65	0.5	0.5	0.5
**Vitamin/trace element premix**	%	1	1	1	1	1	1
**Choline Cl**	%	0.3	0.3	0.3	0.3	0.3	0.3
**Vitamin C, stabilized**	%	0.58	0.029	-	0.58	0.029	-
**Sugar beet pulp**	%	1.3	1.3	1.3	1	1	1
**Soybean oil**	%	1.2	1.2	1.2	0.5	0.5	0.5
**Cholesterol**	%	-	-	-	0.35	0.35	0.35
**Coconut oil, hydrogenated**	%	-	-	-	18	18	18
**Crude protein (=N x 6.25)**	%	17.1	17.1	17.1	16.9	16.9	16.9
**Crude fat**	%	3.8	3.8	3.8	20	20	20
**Crude fiber**	%	19.8	19.9	19.9	11.4	11.4	11.4
**NDF**	%	32.3	32.4	32.4	-	-	-
**Crude ash**	%	7.9	7.9	7.9	6.6	6.6	6.6
**Starch**	%	13.3	13.7	13.7	7.9	7.9	7.9
**Sugar**	%	4	4	4	17.3	17.9	17.9
**Carbohydrates (NfE)**	%	41.1	41.1	41.1	-	-	-
**ME (Atwater)[1]**	MJ/kg	11.2	11.2	11.2	16.8	16.8	16.8

**Kcal Protein**	%	26	26	26	17	17	17
**Kcal Fat**	%	13	13	13	45	45	45
**Kcal CHO**	%	61	61	61	38	38	38
**Fatty Acids (% in diet)**							
**C8:0**	%	-	-	-	1.06	1.06	1.06
**C10:0**	%	-	-	-	0.86	0.86	0.86
**C12:0**	%	-	-	-	8.22	8.22	8.22
**C14:0**	%	0.01	0.01	0.01	3.53	3.53	3.53
**C16:0**	%	0.64	0.64	0.64	2.23	2.23	2.23
**C18:0**	%	0.11	0.11	0.11	2.32	2.32	2.32
**C20:0**	%	0.01	0.01	0.01	0.03	0.03	0.03
**C16:1**	%	0.02	0.02	0.02	0.01	0.01	0.01
**C18:1**	%	0.67	0.67	0.67	0.44	0.44	0.44
**C18:2**	%	1.89	1.89	1.89	0.96	0.96	0.96
**C18:3**	%	0.35	0.35	0.35	0.22	0.22	0.22
**Ascorbic acid**	mg/kg	1740	156	0	1830	143	0

[1] Atwater has been estimated using ME for rats, because it is currently unknown for guinea pigs. Therefore, the ME of high fiber diets for guinea pigs may be inaccurate. Ssniff Spezialdiäten (Soest, Germany) manufactured the feed, and postproduction analysis confirmed the exact vitamin C content. LF: low fat diet, HF: high fat diet, CHO: hydrated carbohydrates, ME: metabolizable energy, NDF: neutral detergent fiber (fiber fraction that is included in the CHO (NfE)), NfE: nitrogen free extracts, VitC: vitamin C.
